# SARS‐CoV‐2–*Streptococcus pneumoniae* Coinfection: A Narrative Review of Pathogenesis, Synergy, and Clinical Impact

**DOI:** 10.1155/ijm/3388334

**Published:** 2026-10-10

**Authors:** Wannaporn Chatwitchanon, Kamontip Kongutsa, Wasu Supharattanasitthi, Teerawit Audshasai

**Affiliations:** ^1^ Department of Microbiology Faculty of Pharmacy Mahidol University Bangkok Thailand mahidol.ac.th; ^2^ Department of Physiology Faculty of Pharmacy Mahidol University Bangkok Thailand mahidol.ac.th; ^3^ Centre of Biopharmaceutical Science for Healthy Ageing Faculty of Pharmacy Mahidol University Bangkok Thailand mahidol.ac.th

**Keywords:** coinfection, COVID-19, SARS-CoV-2, *Streptococcus pneumoniae*

## Abstract

Bacterial coinfection following a respiratory viral infection is one of the main causes of morbidity and mortality during viral pandemics. However, the specific interaction between *S. pneumoniae* and SARS‐CoV‐2 remains scattered and poorly understood. In this narrative review, current evidence regarding the pathogenesis, synergistic effects, and clinical impact of this coinfection was collected and reviewed. Peer‐reviewed literature published between January 2020 and January 2026 was retrieved from PubMed, Scopus, and ScienceDirect, and the evidence is presented separately according to its source, namely, experimental animal‐model data, clinical observations, antimicrobial‐resistance surveillance, and vaccine‐related observational findings. From experimental studies in human angiotensin‐converting enzyme 2 (hACE2) mouse models, direct SARS‐CoV‐2 with *S. pneumoniae* coinfection caused lethal synergy via alveolar macrophage depletion, cytokine storm, and increased mortality. From clinical observations, coinfection was associated with poor outcomes, especially in the elderly and patients with comorbidities, although reported coinfection rates and effect sizes vary considerably between cohorts. Although global nonpharmacologic interventions (NPIs) temporarily reduced pneumococcal transmission, a concerning rise in antimicrobial resistance due to empiric antibiotic use during the pandemic had been observed. Additionally, several observational studies suggest that the 13‐valent pneumococcal conjugate vaccine (PCV13) may be associated with more favorable COVID‐19 outcomes, potentially through the reduction of pneumococcal carriage, although this evidence remains susceptible to residual confounding. Despite evidence of synergism in animal models, clinical mechanisms remain inconclusive. Therefore, future research is required to validate these findings in clinical settings and to provide stronger evidence regarding cross‐protection of the PCV to support valid recommendations.

## 1. Introduction

Throughout history, respiratory viral and bacterial coinfections have significantly contributed to the worldwide disease burden [1]. For instance, RSV, as well as influenza virus, with bacterial infection increases the degree of infection leading to significantly higher morbidity and mortality [2, 3]. COVID‐19, caused by the novel coronavirus SARS‐CoV‐2, emerged in late 2019 and, due to its highly transmissible nature, quickly became a significant global threat [4, 5]. Although COVID‐19′s main symptoms are fever, fatigue, and cough, and most patients show a good prognosis, some may develop severe forms of the disease, such as COVID‐19 pneumonia [6, 7]. In such unfortunate events, the patient′s immune system can become considerably weakened, leaving the body vulnerable to secondary bacterial infections [8, 9].

*Streptococcus pneumoniae* or the pneumococcus, a gram‐positive bacterium, is one of the leading causes of community‐acquired pneumonia, otitis media, sepsis, and meningitis and is known for its disease burden as the leading cause of morbidity and mortality across the globe [10, 11]. In young children, pneumococcus is one of the most common colonizing organisms of the nasopharynx, which can result in either asymptomatic or symptomatic colonization [12]. Even though the highly effective 13‐valent pneumococcal conjugate vaccine (PCV13) has been approved and used for more than a decade, and followed by the recent approval of PCV20 by the US FDA in 2021, sadly, not every country could afford mass‐PCV vaccination into their health scheme [13, 14]. Meanwhile, the nonvaccine serotypes were also shown to emerge due to serotype replacement [11, 15]. Therefore, the threat of pneumococcal infection is still looming.

Several cases of viral infections coinfected with *S. pneumoniae* have been reported, which resulted in significant morbidity and mortality [16–18]. Specifically, pneumococcal coinfection was shown to be correlated with the severity of H1N1 pandemic influenza back in 2009 [19]. The same goes for RSV coinfected with *S. pneumoniae,* which was reported to be associated with increased severity in young children [3, 16]. The incidence of pneumococcal carriage has also been shown to increase during respiratory viral infections [12, 20].

Unlike coinfections with influenza and RSV, information regarding the interaction between SARS‐CoV‐2 and *S. pneumoniae* remains scattered and poorly understood. To capture the available evidence, a structured but nonsystematic search of PubMed, Scopus, and ScienceDirect was performed for records published between January 2020 and January 2026. Search terms combined (“SARS‐CoV‐2” OR “COVID‐19”) with (“*Streptococcus pneumoniae*” OR “pneumococc∗”) and (“co‐infection” OR “coinfection” OR “superinfection” OR “secondary bacterial infection” OR “colonisation” OR “carriage”). Peer‐reviewed original studies reporting experimental (animal or in vitro), clinical, epidemiological, antimicrobial‐resistance, or vaccine‐related data on both pathogens were considered eligible, whereas non‐English records, conference abstracts without an available full text, and studies not addressing both organisms were excluded. Titles and abstracts were screened, and the final set of studies was selected by consensus of the authors. Consistent with its narrative design, this review was not intended to be exhaustive; therefore, no formal risk‐of‐bias, meta‐analytic, or publication‐bias assessment was undertaken, and the synthesis is necessarily dependent on previously published sources. For clarity, this review adopts the following definitions: “Coinfection” refers to concurrent or near‐concurrent detection of both pathogens; “secondary bacterial pneumonia” (or superinfection) refers to pneumococcal disease that arises following, and as a result of, the preceding viral infection [8]; and “colonisation” (or carriage) refers to pneumococcal presence in the upper respiratory tract in the absence of invasive disease [12]. This narrative review synthesizes experimental and clinical evidence on SARS‐CoV‐2–pneumococcal coinfection, aiming to elucidate mechanistic pathways, clinical implications, and areas requiring further investigation. The following section begins by examining the underlying pathogenesis driving this interaction.

## 2. Pathogenesis of SARS‐CoV‐2 and *S. pneumoniae* Coinfection

The pathophysiology of severe SARS‐CoV‐2 pneumonia itself, including direct alveolar injury from viral replication, a dysregulated hyperinflammatory response, diffuse alveolar damage, and a predisposition to secondary bacterial infection, has been thoroughly reviewed by Caliman‐Sturdza et al. [21]. The studies of viral–bacterial coinfection pathogenesis, especially that of the pneumococcal coinfection, were largely coming from influenza A virus (IAV) or RSV as the responsible virus [16–19]. Several mechanisms have been discovered to be linked to pathogenesis, including increased bacterial colonization and invasion, suppression of immunity, and acute lung injury. These pathogenic processes likely act concomitantly or sequentially to facilitate the progression of coinfection [22]. However, the specific pathogenesis of SARS‐CoV‐2 and pneumococcal coinfection remains less thoroughly investigated compared with influenza models. Nevertheless, animal studies demonstrated that such coinfection resulted in severe inflammation and mortality, with several potential pathogenesis [23, 24]. Following are published data of each possible pathogenesis, classified into the same section.

### 2.1. Cytokine Interactions

Several studies observed increases in proinflammatory cytokines such as tumor necrosis factor alpha (TNF‐*α*) and interleukin 6 (IL‐6) during SARS‐CoV‐2 infection, as well as dysregulation of the phagocytes [21, 25–28]. However, experiments using the hACE mouse model with SARS‐CoV‐2 and pneumococci coinfection yielded inconclusive results regarding the timing and intensity of this response. One study indicated a cytokine storm 3 days post‐coinfection, whereas another found no significant difference in proinflammatory cytokines despite using the same infection dose [23, 24]. This inconsistency might highlight the significance of bacterial strain differences. Furthermore, in contrast to IAV–pneumococcal coinfection where Type I interferon (IFN) levels are altered [29, 30], Type I IFN levels did not appear significantly changed during SARS‐CoV‐2–pneumococcal coinfection in a hACE mice model [24]. Consequently, neutralization of IFN signaling, which improves survival in influenza models [31, 32], had no significant outcome on morbidity in SARS‐CoV‐2 coinfection [23]. Therefore, it may be assumed that the pathology of this particular viral–bacterial coinfection may not rely as heavily on these specific cytokine pathways as seen in other coinfections models.

### 2.2. Host Immune Modulation

As shown in Figure 1, one consistent finding across studies is the depletion of alveolar macrophages (AMs), which usually occurs during viral infection and may contribute to an increase in susceptibility to bacterial coinfection [33, 34]. The pathogenesis behind such depletion remains partially unclear, although data suggest that AMs could be infected with the SARS‐CoV‐2 virus, leading to abnormal cytokine production and responsiveness [35–37]. Alternatively, depletion may relate to activation‐induced cell death, as the level of AMs did not proportionately increase despite a dramatic increase in monocyte infiltration [23]. The increasing susceptibility via AM depletion is also consistent with those of influenza–pneumococcal coinfection, which ultimately leads to an overgrowth of invaded pneumococci, causing inflammatory cell infiltration and could potentially be one of the mechanisms underlying both influenza and SARS‐CoV‐2–pneumococcal coinfections [33, 38].

**Figure 1:**
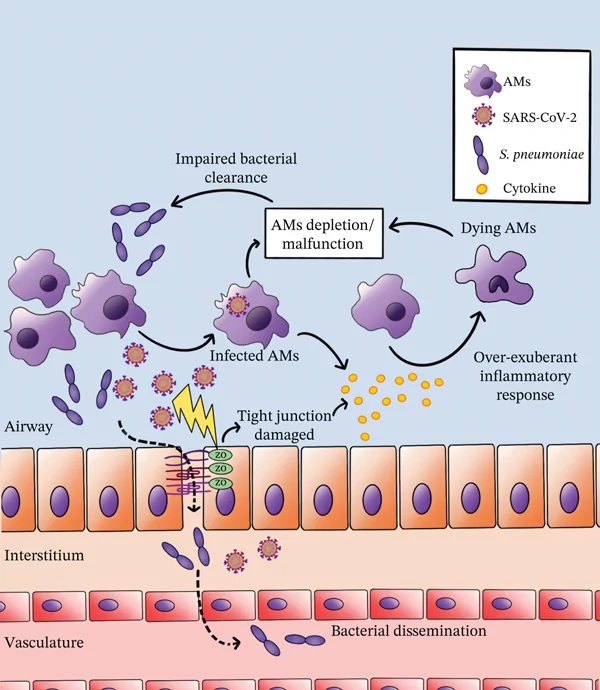
Possible pathogenesis of SARS‐CoV‐2 and pneumococcal coinfection. There are three different mechanisms: (1) SARS‐CoV‐2 interaction with the host tight junction protein ZO1, leading to tight junction damage; (2) infection of AMs by SARS‐CoV‐2, resulting in abnormal cytokine production and responsiveness; and (3) a combination of (1) and (2) causing an overexuberant inflammatory response, leading to AM depletion, which ultimately reduces pneumococcal clearance.

### 2.3. Epithelial Integrity and Viral–Bacterial Synergy

Beyond immune modulation, physical barrier disruption appears to play a critical role. Damage to lung tissue, including hemorrhages, epithelial desquamation, diffuse alveolar damage, and loss of lung tissue structure, was observed in lethal COVID‐19 cases in both mice and humans [21, 39–41]. Specifically, evidence reported the ability of SARS‐CoV‐2 envelope protein to interact with the PDZ Domain 2 of host tight junction protein zona occludens‐1 (ZO1) in infected epithelial cells [42]. As depicted in Figure 1, this interaction may result in tight junction damage and barrier compromise, potentially enhancing viral spreading and promoting bacterial adherence [42]. A similar mechanism could also be seen in IAV or RSV bacterial coinfection [43, 44].

All in all, although the precise molecular mechanisms differ across studies, the consistent finding in animal models points to a lethal synergy driven by two pathogenesis, namely, initial viral‐induced compromise of the alveolar epithelial barrier (via ZO1 interaction) and immune defense (AM depletion), followed by unchecked bacterial proliferation, which ultimately leads to increased mortality (Figure 1). However, the cytokine‐ and IFN‐related impacts are still inconclusive with the current publications and remain to be confirmed.

## 3. Synergism Between SARS‐CoV‐2 and *S. pneumoniae*

### 3.1. Evidence From Animal Models

Although viral–bacterial coinfections are not a common occurrence, pneumococcus is notably reported as one of the infectious agents coinfected with other respiratory viruses, especially IAV [16–19]. Indeed, such bacterial coinfection contributed to a 3.4‐fold increased risk of death when compared with just influenza infection [45]. The pneumococcus was also reported coinfected with respiratory syncytial virus resulting in a higher mortality rate in elderly patients [46]. Such an event was also observed with pneumococcus–SARS‐CoV‐2 coinfection in animal experiments [23, 24].

Two studies depicted the synergy between both pathogens using SARS‐CoV‐2 hACE2 mouse model [23, 24]. Barman et al. observed that although a sublethal dose of either pathogen alone caused minimal weight loss, coinfection caused the experimental mice to lose more than 20% of their body weight and succumb within 5 days of coinfection. As pneumococci are commensal bacteria in the human upper respiratory tract, this study noted that alternating the sequence, infecting with pneumococci first followed by SARS‐CoV‐2 2 days later, also resulted in high mortality (60%), suggesting a bidirectional synergistic relationship [23].

However, the timing of coinfection appears critical, and in a time‐dependent fashion, as demonstrated in Smith et al. study [24]. The study tested the synergy effect of the two pathogens at four different time points, introducing the pneumococcal suspension at Day 1, 3, 5, or 7 postviral infection. Coinfection at Day 1 or 3 did not enhance lethality relative to viral infection alone; the Day 5 group showed a slight, nonsignificant increase in severity, whereas only the Day 7 group was significantly more severe than viral infection alone (*p* = 0.03), reflecting the time‐dependent nature of the coinfection. Similar time‐dependent synergisms were also seen with IAV–pneumococcal coinfection [47]. This delayed susceptibility aligns with the findings discussed in Section 2, specifically, the depletion or dysfunction of AMs, which likely requires several days of viral replication and infection to reach a threshold where they can affect the immune response to secondary bacterial invasion [48].

Indeed, although these models confirm biological plausibility for synergism, critical discrepancies exist. For instance, Barman et al. reported significantly elevated SARS‐CoV‐2 viral loads following coinfection, whereas Smith et al. observed no change in viral replication, finding instead that mortality was driven by bacterial overgrowth [23, 24]. This variability likely stems from several methodological differences between the two studies, which are summarized in Table 1. Specifically, the studies differed in the timing of bacterial challenge and in the dose of the SARS‐CoV‐2 inoculum, with Barman et al. using a higher dose than Smith et al. (1000 PFU vs. 250 PFU), which likely caused greater epithelial injury and immune activation, plausibly explaining why Barman et al. observed a full cytokine storm (elevated IL‐6, TNF‐*α*, MIP‐1*α*), whereas Smith et al. instead saw select cytokines decrease at Day 5. There were also the differences in the pneumococcal serotype employed: The mucoid serotype 3 strain A66.1 (used by Barman et al.) possesses a thicker capsule and is well known to be more virulent and invasive than a laboratory strain such as serotype 2 strain D39 (used by Smith et al.), which could plausibly affect the nature of the coinfection [49]. Moreover, the two studies measured viral load at different time points. Barman et al. sampled 3 days after bacterial challenge, whereas Smith et al. sampled 24 h after bacterial challenge, a difference that possibly explains why Smith et al. found viral loads largely unchanged by coinfection, unlike Barman et al.

**Table 1:** Comparison of experimental designs and key findings from animal‐model studies investigating the synergism between SARS‐CoV‐2 and *S. pneumoniae* coinfection.

Mouse model	Barman et al. (2022)	Smith et al. (2022)
	K18‐hACE2	K18‐hACE2
SARS‐CoV‐2 strain	USA‐WA1/2020	USA‐WA1/2020
Bacterial strain	Serotype 3 (strain A66.1)	Serotype 2 (strain D39)
Route, dose	Intranasal inoculation;	Intranasal inoculation;
	10^3^ PFU SARS‐CoV‐2	250 PFU SARS‐CoV‐2
	10^3^ CFU A66.1	10^3^ CFU D39; total 50 μL
	‐ Total 40 μL in the virus (Day 0) grp.	
	‐ Total 20 μL in the bacteria (Day 0) grp.	
Infection sequence	Bidirectional tested:	Time‐course tested (virus first):
	1. Virus (Day 0) then bacteria (Day 6).	Virus (Day 0) then bacteria added at Day 1, 3, 5, or 7 postinfections.
	2. Bacteria (Day 0) then virus (Day 2).	
Mortality outcome	Sequence‐dependent:	Time‐dependent:
	Virus first group: 100% mortality.	Day 1 or 3 coinfection: lethality was not enhanced when compared with virus infection alone (*p* = 0.73).
	Bacteria first group: 60% mortality.	Day 5 coinfection: more lethal but was not statistically significant (*p* = 0.14).
		Day 7 coinfection: significantly more severe than viral infection alone (*p* = 0.03).

Nevertheless, extrapolating these murine findings to human clinical pathology requires caution. Transgenic hACE2 mice could exhibit nonphysiological expression of hACE2 receptor, like that in the neuroepithelium, potentially leading to neuroinvasion or exaggerated inflammatory responses that do not perfectly mimic human COVID‐19 pneumonia [50]. Therefore, although these animal models provide strong evidence of a lethal synergy, the precise mechanisms in humans are still greatly needed to understand the whole picture of the coinfection. However, such studies are often challenging. Hence, clinical observations serve as an alternative approach to study the synergism of this coinfection in a clinical setting.

### 3.2. Clinical Observations

In a clinical setting, a study by Desai et al. reported that 13% of the 575 patients diagnosed with pneumonia have *S. pneumoniae* and SARS‐CoV‐2 coinfection [51]. The reported coinfection event led to a 15.4% mortality rate; however, this did not differ significantly from the mortality rate of the other pneumonia patients (COVID‐19 positive but *S. pneumoniae* urinary antigen negative), which in fact appeared higher (25.9%). Such difference in mortality rate might stem from the study design in which if the urinary antigen was positive, the patient may likely get tailored antibiotic regimen treatment resulting in a lower mortality rate than the group that got negative in urinary antigen test, resulting in confounding bias [51]. Still, a similar mortality rate (17%) was also observed in other studies by Antón‐Vázquez and Clivillé [52]. However, a more recent study from Morocco by Katfy et al. reported that SARS‐CoV‐2 coinfection with pneumococci significantly increased risk of severe outcomes (OR = 2.861) with high mortality rate of 39.02% compared with 13.9% of the pneumococcal infection only group [53]. The difference between the study designs of each study could be seen in Table 2. This odds ratio should, however, be interpreted with caution, as it was derived from a relatively small number of coinfected patients (*n* = 41 of 120 pneumococcal cases), which limits both the precision of the estimate and its generalizability [53].

**Table 2:** Summary of clinical studies observing the incidence and mortality outcomes of SARS‐CoV‐2 and *S. pneumoniae* coinfection.

Item	Desai et al. (2020)	Anton‐Vazquez and Clivillé (2021)	Katfy et al. (2025)
Study period	April to September 2020	March 4, 2020 to November 4, 2020	January 1, 2020 to December 31, 2022
Base population enrolled	COVID‐19 patients with documented pneumonia in emergency department; u‐Ag testing performed routinely with SARS‐CoV‐2 RT‐PCR.	COVID‐19 admissions with PCR‐confirmed SARS‐CoV‐2; pneumococcal testing performed per guideline/clinician discretion.	All hospitalized patients with confirmed *S. pneumoniae* infection, then stratified by SARS‐CoV‐2 coinfection.
Participants/sample size	Confirmed COVID‐19 cases *n* = 575; u‐Ag performed *n* = 536; coinfected *n* = 68 (13%). Pediatric patients excluded.	Total COVID‐19 positive *n* = 2782; pneumococcal antigen tested *n* = 917; coinfected *n* = 87. Coinfected: mean age 68.	Total pneumococcal infection patients *n* = 120; coinfected *n* = 41. Included patients of all ages.
Diagnostics method	SARS‐CoV‐2: RT‐PCR on nasopharyngeal swab.	SARS‐CoV‐2: PCR on nasopharyngeal swabs.	SARS‐CoV‐2: RT‐PCR on respiratory samples.
	Pneumococcus: u‐Ag performed routinely alongside RT‐PCR.	Pneumococcus: urinary antigen.	Pneumococcus: cultured and confirmed by RT‐PCR.
Main outcome comparison and key finding	Mortality 15.4% (u‐Ag+) vs. 25.9% (u‐Ag−), *p* = 0.09.	30‐day mortality 17% (15/87); no differences vs. pneumococcal‐Ag‐negative COVID patients in ICU admission or 30‐day mortality.	Coinfected vs. noncoinfected: higher ICU admission (53.7% vs. 24.05%) and mortality (39.02% vs. 13.9%); coinfection associated with severe outcome (OR 2.861).

These clinical reports diverge, as Desai et al. found lower mortality in coinfected patients [51], Antón‐Vázquez and Clivillé found no difference in ICU admission or 30‐day mortality [52], and Katfy et al. reported markedly worse outcomes [53]. This divergence likely stems from three factors. First, the studies do not ask the same question; Desai et al. and Antón‐Vázquez and Clivillé began from a COVID‐19 population and asked whether pneumococcal coinfection worsened outcome, whereas Katfy et al. began from a pneumococcal population and asked the converse, so their reference groups and effect estimates are not directly comparable. Second, the studies differ in diagnostic method: Urinary antigen testing is highly sensitive and can remain positive despite prior antibiotic therapy, so a positive result need not reflect active invasive infection, whereas the culture with RT‐PCR confirmation used by Katfy et al. could be interpreted as an ongoing invasive disease [54]. Antigen‐based cohorts may therefore include already‐treated or noninvasive cases that dilute any outcome association, which may partly explain why the culture‐based study reported worse phenotypes. Lastly, due to confounding by treatment, antigen‐positive patients may have received earlier targeted antibiotics, lowering apparent mortality. Weighing these factors, the better controlled culture‐based evidence suggests coinfection is associated with more severe disease, though overall certainty remains low given the small, heterogeneous, and largely retrospective single‐center studies available. Nevertheless, the inconclusive nature of the synergistic properties of this coinfection in a clinical setting may require further studies or a larger cohort with comparable control groups to better understand its impact.

This gap between cleared animal‐model synergy and the more variable clinical picture is expected. Murine studies used fixed high‐dose inocula, controlled timing, invasive serotypes, and highly susceptible transgenic hosts to reveal worst‐case mechanistic synergy [23, 24], whereas in patients, this effect is likely attenuated by pre‐existing immunity, vaccination, antibiotics, comorbidities, and mixed colonizing/invasive strains. The two evidence types are therefore complementary; animal data establish that lethal synergy can occur and how, whereas clinical data indicate that its real‐world impact, though likely harmful, is more modest and heterogeneous.

## 4. SARS‐CoV‐2 Coinfected With *S. pneumoniae* in Clinical Setting

### 4.1. Lower Incidence due to Nonpharmacologic Interventions (NPIs)

NPIs are strategies implemented to control the spread of infectious diseases without the use of medications or vaccines, such as social distancing, wearing personal protective equipment (PPE), hand hygiene, and enforced lockdown [55]. Such interventions were made famous globally during the recent COVID‐19 pandemic.

Although intended for the virus, these measures significantly disrupted the transmission dynamics of respiratory bacteria as shown by several studies [56–59]. One cross‐sectional study conducted in the United Kingdom focusing on children under 5 years old measured the prevalence of pneumococci in the nasopharynx [56]. Interestingly, the carriage prevalence during pre‐NPI period versus during NPI period showed a striking reduction (32% vs. 19%, respectively) suggesting that NPIs effectively reduced the transmission of the pneumococci in the community. Similarly, surveillance data from Israel and Canada reported reductions in invasive pneumococcal disease (IPD) incidence following NPI implementation [57–59]. The enforcement of NPI also lowered the incidence of other respiratory infections, including influenza and RSV as well [57, 60].

However, such reduction was fleeting as the carriage prevalence increased again after the enforced NPI period was over [56] and similarly with the rate of IPD, which rapidly rose after as well [58, 59]. This development suggests that NPIs may suppress transmission but have a little effect in reducing the community reservoir. Furthermore, the reported reduction in IPD cases may be confounded by altered healthcare‐seeking behavior as the fear of contracting COVID‐19 likely deterred individuals from visiting hospitals, leading to an underdiagnosis of milder, self‐limiting invasive pneumococcal cases [56, 61].

### 4.2. Coinfection in Various Age Groups

Indeed, as one′s age progresses, their immune system will deteriorate gradually; such event is referred to as immunosenescence [62]. Immunosenescence affects both innate and adaptive immunity, which limits the response to pathogens as a result [62, 63]. Therefore, aging individuals are at higher risk of both single and coinfection as numerous studies observed [64–66]. The combination of immunosenescence and the SARS‐CoV‐2–induced depletion of AMs renders elderly patients compromised hosts, significantly heightening their susceptibility to secondary bacterial coinfection, including but not limited to *S. pneumoniae* [24, 33, 64]. Alas, to the best of our knowledge, no study has performed a statistical test to determine whether SARS‐CoV‐2 and pneumococcal coinfection is more prevalent than other bacterial coinfections, nor directly compared incidence rates across different groups of patients.

Most epidemiological and surveillance studies focus broadly on SARS‐CoV‐2 and any bacterial coinfection. Here, we present a collection of studies specifically highlighting the incidence of pneumococcal coinfection in the elderly (mean or median age 60 years) (Table 3). However, these data should be interpreted with caution. Because of the lack of controlled studies or meta‐analyses, significant heterogeneity between studies, ranging from clinical settings to diagnostic methods, can lead to divergent outcomes. For instance, Contou et al. focused primarily on critically ill patients requiring mechanical ventilation, a population more prone to ventilator‐associated pneumonia (VAP) than to coinfection with community pathogens like *S. pneumoniae* [67]. Similarly, the outlier reported by Moreno‐García et al., where *S. pneumoniae* accounted for 79% of all bacterial coinfections, likely stems from the use of the urinary antigen test. This method is far more sensitive than standard culture techniques, which are often confounded by empirical antibiotic use, thereby leading to lower reported incidence in other studies [68]. On the contrary, Thelen et al. reported pneumococcal coinfection in only 3.3% of cases, which attributed largely to culture contamination by coagulase‐negative staphylococci rather than to a true absence of the pathogen [69]. Taken together, the incidence across these elderly cohorts spans roughly 3.3%–79%, a big variation that reflects differences in detection method and clinical setting than a genuine epidemiological variation.

**Table 3:** Incidence of pneumococcal coinfections with SARS‐CoV‐2 in the elderly with a mean or median age of 60 years or greater.

Study name	Study design and population	Key findings	Remarks
Contou et al. [67]	Case‐control	Incidence: 6 cases	Focused on the ICU setting; hence, it reported more ventilator‐associated pneumonia (VAP) than community‐acquired infections.
	Median age: 61	(23% of bacterial coinfections)	
Moreno‐García et al. [68]	Case‐control	Incidence: 81 cases	The exceptionally high rate (79%) is possibly due to the use of urinary antigen tests, which detect bacteria even after empirical antibiotic treatment, unlike culture‐based methods.
	Median age: 64; *n* = 102	(79% of bacterial coinfections)	
Hedberg et al. [16]	Case‐control	Incidence: 13 cases	Identified *S. pneumoniae* as the most common bacterium coinfecting with SARS‐CoV‐2, followed by *S. aureus*.
	Median age: 62; *n* = 46	(28% of bacterial coinfections)	
Thelen et al. [69]	Case‐control	Incidence: 2 cases	The low percentage of pneumococcal coinfection was attributed to contamination by coagulase‐negative staphylococci (85.8%). However, *S. pneumoniae* remained the most common pathogen identified.
	Median age: 70; *n* = 61	(3.3% of bacterial coinfections)	
Garcia‐Vidal et al. [70]	Median age: 63; *n* = 74	(16.21% of bacterial coinfections)	This study classified coinfection by the timing of infection (at diagnosis vs. hospital‐acquired), in which *S. pneumoniae* ranked first in the “infection at COVID‐19 diagnosis” group (12 out of 30 cases).
Gudiol et al. [71]	Prospective multicenter cohort study	Incidence: 9 cases	This study classified coinfection by organ system; *S. pneumoniae* was the most common pathogen in patients with respiratory tract infections (9 out of 21 cases).
	Median age: 67.5; *n* = 54	(16.7% of bacterial coinfections)	
Cheng et al. [72]	Retrospective multicenter study	Incidence: 11.5% of bacterial coinfections	This study focused on coinfection during the Omicron outbreak; *S. pneumoniae* was still reported as the primary causative agent.
	Median age: 75		

In sharp contrast, the pediatric population exhibits a paradoxically lower rate of bacterial coinfection. A large meta‐analysis of 17,588 children with COVID‐19 found a bacterial coinfection rate of only 4.7%, compared with 15.9% in adults [73, 74]. A more recent surveillance study also reported coinfection of respiratory viruses and pneumococci to be mostly predominated by other respiratory viruses like RSV instead of SARS‐CoV‐2 [75].

Unlike adults, this protection is likely linked by the lower expression of ACE2 receptors in pediatric epithelial cells. Because ACE2 is the entry receptor for SARS‐CoV‐2, lower expression results in reduced viral load and less severe initial lung damage, thereby preserving the lung environment and preventing the opportunistic bacterial invasion seen in adults [76]. Nevertheless, when coinfection does occur in children, *S. pneumoniae* remains one of the leading pathogens, potentially leading to severe complications like acute respiratory distress syndrome (ARDS) [73].

### 4.3. Coinfection in Patients With Comorbid(s)

A study stratifying COVID‐19 patients based on obesity and concurrent comorbidities (including diabetes, hypertension, and COPD) revealed that over 90% of patients in both groups presented with bacterial coinfection, frequently involving *S. pneumoniae* [77]. Similarly, a study from China reported that patients with diabetes exhibited a significantly higher incidence of pneumococcal coinfection [72].

Indeed, diabetes, chronic cardiovascular disease, COPD, asthma, and chronic kidney disease are established risk factors for *S. pneumoniae* coinfection [78]. In patients with uncontrolled diabetes, hyperglycemic conditions lead to significant immune impairments, including decreased neutrophil and macrophage function. This impairs chemotaxis, adherence, and phagocytosis, consequently increasing the risk of pneumococcal coinfection [79]. Similarly, patients with COPD exhibit increased ACE2 expression alongside reduced innate and adaptive immune responses and impaired endothelial cell function [80]. Additionally, hypersecretion of mucus in the respiratory tract is common in these patients, creating a favorable environment for *S. pneumoniae* to proliferate within mucus plugs [81]. This ultimately renders such patients highly susceptible to coinfection with SARS‐CoV‐2 and *S. pneumoniae* [80]. A similar reduction in immune competency is observed in patients with chronic kidney disease and chronic cardiovascular disease, thereby increasing the risk of bacterial coinfection [82–84]. Regarding obesity, this condition drives the excessive production of proinflammatory cytokines such as IL‐6, TNF‐*α*, IL‐1*β*, and monocyte chemoattractant protein‐1 (MCP‐1); collectively, these markers may dysregulate immune function and trigger pyroptosis of lung cells, thereby heightening the risk of bacterial coinfection in these patients [77, 85]. Likewise, patients with cancer whose immune systems were weakened due to several factors whether from their ages, the cancer itself, or the side effects of the medication taken to battle against such deleterious disease are also prone to SARS‐CoV‐2–bacterial coinfection [71]. The incidence of *S. pneumoniae* coinfection in such a group of patients was also remarkably high when compared with other respiratory tract infections, constituting around 40% of all respiratory tract bacterial coinfection in COVID‐19 patients with cancer background [71].

However, just as before, the association between these comorbidities and coinfection rates must be interpreted with caution because of several confounding factors. For instance, age serves as a major confounder; because the prevalence of these comorbidities naturally increases with age, it becomes difficult to separate the specific pathophysiological impact of a comorbidity from the general effects of immunosenescence. Surveillance bias also presents a significant confounding factor, as patients with multiple comorbidities typically present with more severe COVID‐19 symptoms [86]. Consequently, they are more likely to undergo extensive microbiological testing compared with healthier individuals, which potentially skews the reported incidence rates. All in all, dedicated epidemiological studies or meta‐analyses focusing specifically on *S. pneumoniae* and SARS‐CoV‐2 are still needed to capture the whole picture of the different factors affecting this coinfection.

## 5. Antimicrobial Resistance (AMR) and Vaccine Effect

### 5.1. AMR of *S. pneumoniae* During the Pandemic

AMR is one of the concerning healthcare problems long before the SARS‐CoV‐2 pandemic [87, 88]. Although several studies observed the rise in AMR cases among COVID‐19 patients [89, 90], most focused on notorious resistance strains such as MRSA and carbapenem‐resistant Enterobacteriaceae [89–92]. However, some studies also included data with *S. pneumoniae*, warranting a discussion.

#### 5.1.1. Global Trends in Pneumococcal Resistance

Although the cases of IPD were lower due to nonpharmacologic interventions (NPIs) [56–58], surveillance data show an increase in AMR among circulating *S. pneumoniae* strains [93].

According to the routine surveillance data reported to the European Centre for Disease Prevention and Control, it appears that the resistance rate of penicillin group slightly increased from 12.2% in 2019 to 15.6% in 2020. The same trend was observed for the macrolides group as well, where the resistance rate increased from 14.5% in 2019 to 16.9% in 2020 [93, 94]. A nationwide surveillance study from Spain also shows an increase in resistance rates for several antibiotics from 2019 to 2020, namely, amoxicillin (33% in 2019 vs. 36% in 2020), cefixime (68% vs. 72%), cefpodoxime (50% vs. 56%), and erythromycin (55% vs. 59%), whereas the antibiotics that characterized the lowest resistant rate during the study period were cefditoren (< 0.4%) and cefotaxime (< 5%), making them a viable option for treating pneumococcal infection [95].

On the same note, studies focusing on coinfection of SARS‐CoV‐2 and *S. pneumoniae* also demonstrate similar resistance burden. In Iraq, *S. pneumoniae* coinfected with SARS‐CoV‐2 demonstrated high‐level resistance to ceftriaxone (MIC = 64) [96]. In Spain, an analysis of 57 pneumococcal isolates from COVID‐19 patients revealed that although penicillin susceptibility remained high (MICs ≤ 2 mg/L), resistance was notable for other classes: 26.3% for macrolides, 22.8% for tetracycline, and 15.8% for co‐trimoxazole [59].

#### 5.1.2. Drivers of Resistance

One of the possible reasons that cause an increase in AMR strains of pneumococci might stem from the behavioral changes regarding healthcare‐seeking behaviors of the masses. With the pandemic ongoing, many individuals avoid or delay seeking medical care in fear of contracting COVID‐19 in healthcare settings, which in turn leads to inappropriate or prolonged antibiotic use when they do seek care, thus contributing to resistance [93]. There was also a notable rise in the use of antibiotics as an empirical therapy, especially macrolides, as many practitioners prescribed empirically for pneumonia in fear of atypical bacteria, which in turn may have contributed to the selection pressure that fosters antibiotic‐resistant strains of pneumococci [97–99].

There was also report of the link between non‐PCV13 serotypes and antibiotic resistance during pandemic era. Serotypes 24F and 11A appear to be the most concerning out of other non‐PCV13 serotypes, with serotype 24F′s tendencies to resist both penicillin and erythromycin and 11A′s ability to produce biofilms, which contributed to its invasiveness and resistance [95, 100, 101]. Both serotypes′ prevalence has also been increasing in both pediatric and adult populations in the country in which PCV13 has been introduced [102, 103].

Ultimately, during the chaotic pandemic situation, the focus on managing COVID‐19 and the patients′ condition may have diverted attention and resources from performing proper antibiotic stewardship as under normal circumstances, thereby exacerbating the problem of resistance [93].

### 5.2. Vaccine Effect

Although both PCV13 and mRNA SARS‐CoV‐2 vaccines have demonstrated efficacy individually in several studies [104–106], the potential efficacy of these vaccines in the context of coinfection is not yet well understood. Proving a direct connection in a clinical setting is challenging; therefore, using a murine model could roughly give us an idea reflecting such a situation.

After immunizing the mice with a full regimen of Pfizer mRNA SARS‐CoV‐2 vaccine and Prevnar13 vaccine, the same mice were subjected to SARS‐CoV‐2 challenge followed by *S. pneumoniae*. In contrast to the unvaccinated mice, all of the vaccinated group showed 100% survival after challenge, whereas the others fell within 11 days after the challenge. A significant decrease in proinflammatory cytokine levels and immune cell infiltration was also observed in vaccinated mice. Furthermore, vaccinated mice demonstrated a recovery of AM levels after challenge, which in turn lowered the bacterial load significantly [23].

Even so, in clinical settings, several studies have reported serotype 3 as a leading cause of coinfection, followed by 19A [53, 59]. Even though these two serotypes are included in PCV13, the effectiveness of the vaccine against them is somewhat limited [107]. This is especially true for serotype 3, given its ability to release capsular polysaccharide during growth, which may hinder anti‐Type 3 capsular antibody‐mediated protection [108].

Interestingly, observational data have suggested a correlation between PCV13 vaccination and improved COVID‐19 outcomes, although this should be interpreted with care [109, 110]. One of the cohort studies included a total of 531,033 individuals aged 65 years or older. Using adjusted hazard ratios, the study highlighted that PCV13 recipients had a significantly reduced risk of severe COVID‐19 outcomes compared with those who did not receive the vaccine [109]. The other cohort study, using adjusted hazard ratios, also concluded that those who received PCV13 experienced a modestly lower risk of confirmed SARS‐CoV‐2 infection compared with those who did not [111].

These findings suggest that preventing pneumococcal carriage could potentially influence SARS‐CoV‐2 susceptibility. Studies conducted in the early COVID‐19 pandemic period found an association between the two organisms; specifically, individuals who carried pneumococcus had higher rates of SARS‐CoV‐2 acquisition and longer durations of viral shedding compared with the noncarriers [109, 112]. This carriage interaction influence is supported by the fact that the reduced risk of SARS‐CoV‐2 infection seen in PCV13 vaccinated individuals was not observed in the people who had only been vaccinated with pneumococcal polysaccharide vaccine (PPSV23), which typically does not affect pneumococcal carriage [109].

Moreover, the potential protection of PCV13 against SARS‐CoV‐2 infection in vaccinated individuals seems to be lessened by recent prior antibiotic use, supporting the relationship between carriage pneumococci and SARS‐CoV‐2 [109]. Interestingly, lower levels of SARS‐CoV‐2–specific memory B cell and salivary IgA were also observed in pneumococcal‐carriage subjects compared with the noncarriage subjects, further suggesting the positive influence the bacteria had on SARS‐CoV‐2 [110, 113]. Similar interactions were also observed with other respiratory viruses [114–116].

Even though most data on the effectiveness of PCV13 against SARS‐CoV‐2 infection stem from observational cohort studies rather than a head‐to‐head clinical trial, the population in which PCV13 as a vaccine gives the most benefit, including the elderly, individuals with obesity, and those with diabetes, is also the same risk group for severe SARS‐CoV‐2 infection [117–119]. Therefore, vaccination could prove beneficial to susceptible groups.

However, even with all these findings, the current evidence linking pneumococcal vaccination to SARS‐CoV‐2 protection is primarily epidemiological and associative. Mechanistic evidence in humans remains lacking, and although animal models demonstrate synergy and vaccine cross‐protection, these results cannot translate directly to clinical efficacy. Pneumococcal vaccination should therefore be regarded as, at most, associated with rather than proven to be protective against SARS‐CoV‐2 outcomes. Further investigation and/or meta‐analysis is required to definitively determine whether the observed benefits reflect genuine biological cross‐protection or residual confounding in vaccinated populations, such as healthy‐vaccinee effects and differences in health‐seeking behavior [120].

## 6. Conclusion

The interplay between SARS‐CoV‐2 and *S. pneumoniae* has underscored significant challenges in managing respiratory infections during the COVID‐19 pandemic. Documented cases indicate that *S. pneumoniae* is one of the frequently reported bacterial pathogens coinfecting COVID‐19 patients, particularly in association with severe outcomes in the elderly and individuals with underlying comorbidities. Although precise clinical pathogenesis is difficult to confirm in humans, hACE2 mouse models have demonstrated a lethal synergism largely driven by the depletion of AMs and severe inflammation. The pandemic also introduced dual pressures on pneumococcal dynamics: NPIs temporarily reduced IPD, yet rampant empirical antimicrobial use sparked concerning rises in resistant *S. pneumoniae* strains. In contrast, observational data suggest that pneumococcal vaccination (e.g., PCV13) may offer some associated benefits against SARS‐CoV‐2 infection by reducing baseline nasopharyngeal colonization.

However, current conclusions are limited by the scarcity of clinical data that focus only on the relationship between the two pathogens and also by the significant heterogeneity between experimental animal models and complex clinical settings. As a narrative rather than systematic review, this work summarizes the available evidence but does not statistically pool it; it does not provide a quantitative meta‐analytic estimate, formally quantify between‐study heterogeneity, or assess publication bias, and it necessarily relies on previously published sources. These limitations should be borne in mind when interpreting the associations described above. Future research may prioritize addressing these gaps by translating in vivo findings into clinical settings, including aspects of their pathogenesis and synergism, as well as the benefits of PCV against SARS‐CoV‐2 infection, which should be tested through robust randomized controlled trials. This narrative serves as a link between scattered information about SARS‐CoV‐2 and *S. pneumoniae* coinfection, which strongly highlights the need for continued research and proactive measures to address the challenges posed by these coinfections.

## Author Contributions

Teerawit Audshasai and Wasu Supharattanasitthi formulated the idea, participated in overall supervision, and were responsible for revising this manuscript. Wannaporn Chatwitchanon and Kamontip Kongutsa contributed equally to the initial drafting and literature review. Wannaporn Chatwitchanon and Kamontip Kongutsa contributed equally to this work and co‐first authors.

## Funding

No funding was received for this manuscript.

## Disclosure

All authors have given final approval of the version to be published and agreed to take responsibility for all aspects of the work, ensuring that any questions regarding the accuracy or integrity of any part of the work are properly addressed and resolved.

## Conflicts of Interest

The authors declare no conflicts of interest.

## Data Availability

Data sharing is not applicable to this article as no datasets were generated or analyzed during the current study.
